# Design and methodology of the SARCopenia trajectories and associations with adverse clinical outcomes in patients on HemoDialysis: the SARC-HD study

**DOI:** 10.1186/s12882-023-03168-4

**Published:** 2023-08-15

**Authors:** Marvery P. Duarte, Marina S. Pereira, Victor M. Baião, Fábio A. Vieira, Maryanne Zilli Canedo Silva, Rodrigo R. Krug, Antônio J. Inda-Filho, Aparecido P. Ferreira, Ricardo M. Lima, Carla Maria Avesani, Otávio T. Nóbrega, Maycon M. Reboredo, Heitor S. Ribeiro, on behalf of the SARC-HD Study Group

**Affiliations:** 1 Present Address: Faculty of Health Sciences, University of Brasilia, Brasília, Brazil; 2 Pulmonary and Critical Care Division, University Hospital of Federal University of Juiz de Fora, Juiz de Fora, Minas Gerais, Brazil; 3 IdealCor Physiotherapy, Brasília, Brazil; 4 Internal Medicine Department, Botucatu Medical School, São Paulo State University, UNESP, Botucatu, Brazil; 5 University of Cruz Alta, Cruz Alta, Brazil; 6 Interdisciplinary Research Department, University Center ICESP, Brasilia, Brazil; 7 Faculty of Physical Education, University of Brasilia, Brasília, Brazil; 8 Department of Clinical Science, Technology and Intervention, Division of Renal Medicine and Baxter Novum, Karolinska Institute, Stockholm, Sweden; 9 School of Medicine, University of São Paulo, São Paulo, Brazil

**Keywords:** Chronic kidney disease, Dialysis, Sarcopenia, SARC-F, Physical function

## Abstract

**Background:**

Sarcopenia has been associated with adverse outcomes in patients with chronic kidney disease (CKD), particularly in those undergoing hemodialysis (HD). However, the trajectories across sarcopenia stages, their determinants, and associations with adverse clinical outcomes have yet to be comprehensively examined.

**Methods:**

The SARC-HD is a multicenter, observational prospective cohort study designed to comprehensively investigate sarcopenia in patients on HD. Eligibility criteria include adult patients undergoing HD for ≥ 3 months. The primary objective is to investigate the trajectories of sarcopenia stages and their potential determinants. Secondary objectives include evaluating the association between sarcopenia and adverse clinical outcomes (*i.e.*, falls, hospitalization, and mortality). Sarcopenia risk will be assessed by the SARC-F and SARC-CalF questionnaire. Sarcopenia traits (*i.e.*, low muscle strength, low muscle mass, and low physical performance) will be defined according to the revised European Working Group on Sarcopenia in Older People and will be assessed at baseline and after 12 follow-up months. Patients will be followed-up at 3 monthly intervals for adverse clinical outcomes during 24 months.

**Discussion:**

Collectively, we expect to provide relevant clinical findings for healthcare professionals from nephrology on the association between sarcopenia screening tools (*i.e.*, SARC-F and SARC-CalF) with objective sarcopenia measurements, as well as to investigate predictors of trajectories across sarcopenia stages, and the impact of sarcopenia on adverse clinical outcomes. Hence, our ambition is that the data acquired from SARC-HD study will provide novel and valuable evidence to support an adequate screening and management of sarcopenia in patients on HD.

**Supplementary Information:**

The online version contains supplementary material available at 10.1186/s12882-023-03168-4.

## Background

Sarcopenia and its related traits (*i.e.*, low muscle strength, low muscle mass, and low physical performance) are prevalent conditions and have been associated with a wide range of adverse outcomes, including mortality, in patients with chronic kidney disease (CKD), particularly in those undergoing hemodialysis (HD) [1–4]. Although the incidence of sarcopenia is more common in older people, CKD *per se* is an important risk factor for accelerated aging. The multiple etiologic factors caused by CKD, such as uremic state, metabolic acidosis, inflammation, malnutrition, and sedentary behavior may lead to notorious changes in physical function and nutritional status according to the progress of CKD, reflecting a *continuum* of disease-related impacts, which may lead to the development of sarcopenia and its related traits [5, 6].

Previous observational studies have shown that sarcopenia is a condition of dynamic nature, which may worsen or regress over time [7–9]. In summary, these studies indicate potential determinants of sarcopenia progression in older people, such as physical activity levels, nutrition, cognitive function, body mass index, smoking, multimorbidity, male gender, and age. Interestingly, Trevisan et al. showed that higher physical activity levels and preservation of cognitive status were associated with the reversibility from probable sarcopenia to a non-sarcopenia stage [8]. On the other hand, despite the growing interest of the scientific nephrology community regarding CKD-related sarcopenia [6, 10, 11], to the best of our knowledge, no previous study investigated the incidence and trajectories of sarcopenia stages in a longitudinal study with periodical reassessment and their potential determinants in patients on HD. Therefore, from the public health, clinical research, and practical applicability perspectives, further knowledge of the determinants associated with temporal changes of sarcopenia stages is important and may help in the development of therapeutic interventions with greater effectiveness to prevent or counteract the progression of sarcopenia, which can ultimately improve the prognoses and quality of life in this population [12].

In clinical practice, screening tools to identify patients at risk of sarcopenia have been endorsed, including the SARC-F questionnaire [13]. Additionally, indirect markers of muscle mass to improve the accuracy of SARC-F have been proposed, such as calf circumference, referred to as SARC-CalF [14]. Nevertheless, there is limited information regarding the potential application of SARC-F and SARC-CalF in the CKD population and their association with adverse clinical outcomes, especially in patients undergoing HD [15–20]. Altogether, these previous works provide the support that SARC-F and SARC-CalF are relevant in clinical practice to screen sarcopenia risk and are also associated with objective measures of sarcopenia traits such as strength, muscle mass, and performance. Despite the meaningful information, previous studies had relatively small sample sizes and were single-center, requiring a representative sample to robust conclusions. Moreover, the association of SARC-F and SARC-CalF with the occurrence of falls, hospitalization, and mortality remains underexplored in this population.

To address these knowledge gaps, the *SARCopenia trajectories and associations with adverse clinical outcomes in patients on HemoDialysis* (**SARC-HD**) Study was designed to investigate the trajectories of sarcopenia stages and its determinants, as well as the association between sarcopenia and adverse clinical outcomes in patients on HD.

## Methods

The present protocol is reported using adapted versions of the Standard Protocol Items: Recommendations for Interventional Trials (SPIRIT) and Strengthening the Reporting of Observational Studies in Epidemiology (STROBE) guidelines [21, 22].

### Study design and setting

**Fig. 1:**
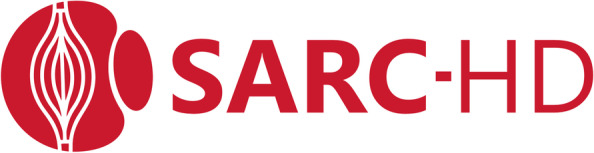
The SARC-HD study logo. *Art by Rebecca Hopkinson*

**Fig. 2:**
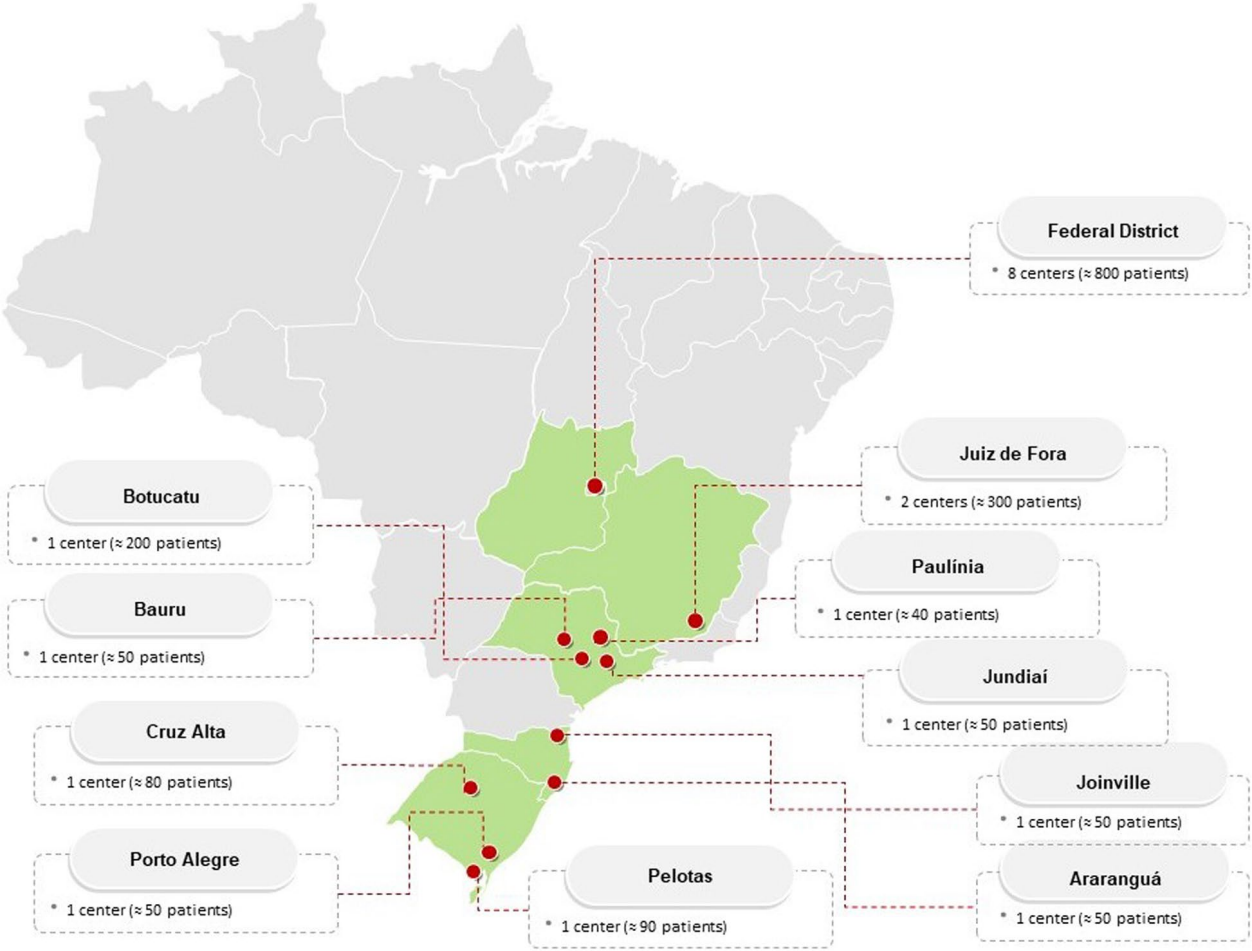
Map of Brazil with the dialysis centers included in the SARC-HD Study

The SARC-HD is a multicenter and observational prospective cohort study that will enroll patients undergoing HD across dialysis centers in Brazil (Fig. 1). Dialysis centers are based in the South (Joinville and Araranguá – Santa Catarina State, Porto Alegre, Pelotas and Cruz Alta – Rio Grande do Sul State), Southeast (Juiz de Fora – Minas Gerais State, Botucatu, Bauru, Jundiaí, and Paulínia – São Paulo State) and Midwest (Brasília – Federal District) regions (Fig. 2). A full list of the SARC-HD study center investigators and coordinators may be seen in Supplementary Material 1).

### Study objectives

#### Primary objective

The primary objective of the SARC-HD study is to investigate the trajectories of sarcopenia stages and examine their potential determinants in patients undergoing HD.

#### Secondary objectives

The key secondary objectives are to investigate the association between sarcopenia traits (*i.e.*, low muscle strength, low muscle mass, and low physical performance) with adverse clinical outcomes (*i.e.*, falls, hospitalization, and mortality).

Other objectives are:

- To investigate whether the insertion of other anthropometric measurements into the SARC-F, such as mid-arm muscle circumference, would improve the predictive ability of sarcopenia screening.
- To evaluate the association between SARC-F, SARC-CalF and 7-point subjective global assessment (7p-SGA) with sarcopenia-related markers, (e.g., bioimpedance variables, laboratory parameters, and anthropometric measuresment).
- To identify the sensitivity and specificity of the proposed cut-off values of the SARC-F (≥ 4) and SARC-CalF (≥ 11) for detecting sarcopenia.
- To compare the performance among SARC-F, SARC-CalF, and 7p-SGA as predictors of adverse clinical outcomes.
- To investigate the performance of the SARC-F, SARC-CalF and 7p-SGA for monitoring longitudinal changes of objective sarcopenia-related measurements (*e.g*., physical function and muscle mass).
- To investigate the prevalence of sarcopenic obesity and associated factors.

### Hypotheses

#### Primary hypothesis

Across 12 follow-up months, we will observe the trajectories among sarcopenia stages mediated in greater magnitude by physical function compared to muscle mass. Factors such as advanced age, female gender, low physical activity levels, poor nutritional status, impaired cognitive status, longer HD vintage, type of treatment, and laboratory variables, such as creatinine and albumin, will be predictors of these trajectories.

#### Secondary hypothesis

Physical function components of sarcopenia will be mostly associated with adverse clinical outcomes than sarcopenia confirmed and muscle mass solely in patients on HD. Also, screening tools for the risk of sarcopenia such as the SARC-F questionnaire and SARC-CalF will be associated with objective measurements of sarcopenia. Nonetheless, the SARC-CalF will show a stronger agreement with sarcopenia traits and better performance to predict adverse clinical outcomes in this population than the SARC-F.

### Study population and eligibility criteria

Patients receiving HD across dialysis centers are eligible for inclusion. The SARC-HD study aims to enroll real-world patients without stringent eligibility criteria. Table 1 provides detailed inclusion and exclusion criteria.

**Table 1:** Inclusion and exclusion criteria for the SARC-HD study

**Eligibility criteria**
1. Aged ≥ 18 years
2. Hemodialysis treatment ≥ 3 months
3. Provided free and informed consent
**Exclusion criteria**
1. Presence of musculoskeletal or other abnormalities that impair examining physical function
2. Medical contraindication for carrying out the battery of physical function tests
3. Uncontrolled heart disease, and very recent hospitalization during baseline assessment (≤ 1 month)
4. Pregnant or breastfeeding

### Outcomes measures

#### Primary outcomes

Trajectories over sarcopenia stages (*i.e.*, none, probable, confirmed, and severe) according to the revised European Working Group on Sarcopenia in Older People (EWGSOP2) from baseline to 12 follow-up months. To explore the adverse clinical outcomes regarding muscle mass, we will also consider probable sarcopenia as low muscle mass, which was recommended in the first version of EWGSOP [23].

#### Secondary outcomes

The secondary outcomes will include:

1. Falls: a fall will be defined as an event that results in a person coming to rest inadvertently on the ground or floor or other lower level. Therefore, we will ask the patients "In the past 3 months, have you had any falls including a slip or trip in which you lost your balance and landed on the floor or ground or lowest level?" [24]. When the patient reports a fall experience during this period, the number, circumstances, and details of any injuries caused by the fall will be recorded.
2. Hospitalization: we defined hospitalization as an unintentional hospital stay that included a minimum of 12 hours in the hospital during follow-up [25]. Access-related hospitalizations will not be considered.
3. Mortality: all-cause and cardiovascular mortality will be obtained from medical records. If necessary, the patient’s family will be contacted to give information regarding the cause and date of death.

All secondary outcomes will be assessed every three months over the 24 follow-up months. The survival time will be measured in months and defined as the period between the date of enrollment and the occurrence of an adverse event. We will censor follow-up time for patients who change peritoneal dialysis modality, loss to follow-up, undergone kidney transplantation, transfer of center, or end of the study.

### Other variables

Sociodemographic, socioeconomic, laboratory variables, dialysis-related parameters and prescriptions, comorbid conditions, anthropometric and body composition, physical activity levels, cognitive status, exercise rehabilitation intervention and nutritional status will be included (more detail in “Data Collection Methods”).

### General procedures

#### Recruitment of dialysis centers

Recruitment of dialysis centers took place in advance during online meetings (from January to August 2022). The principal (HSR) and leading investigators (MPD and MMR) invited members of the Brazilian Group of Nephrology Rehabilitation [26]. Noteworthy, most dialysis centers are coordinated by experienced researchers and have already implemented systematic physical function and body composition assessments.

#### Recruitment of patients

The period of patients' recruitment and baseline evaluation will take place from October 2022 to May 2023. Each dialysis center will adopt the best strategy of recruitment, considering that the clinical and research routines can be clearly distinct. The SARC-HD study is comprised of three phases: baseline assessment, 12 months (reassessment for sarcopenia traits) and 24 months of follow-up.

**Fig. 3:**
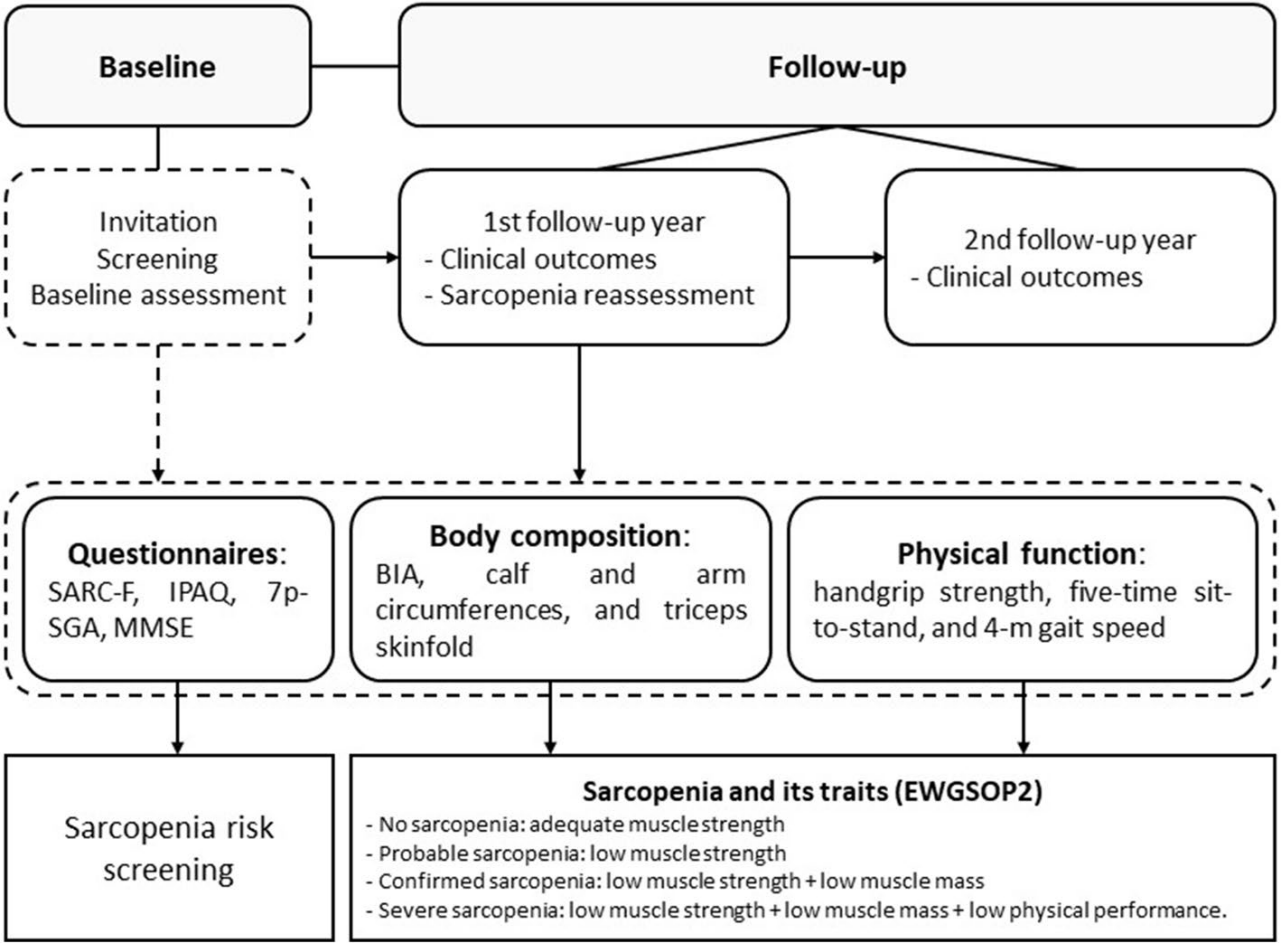
Schematic representation of the SARC-HD study. Abbreviation: *7p-SGA*, 7-point subjective global assessment, *BIA* Bioimpedance analysis, *IPAQ* International physical activity questionnaire, *MMSE* Mini-mental state exam

To enroll the largest number of patients, invitations will be carried out individually by the local research team. Patients who show initial interest will undergo screening to assess for inclusion criteria and sign the informed consent form. A schematic representation of the SARC-HD study is shown in Fig. 3.

### Data collection methods

The SARC-HD study data collection schedule is shown in Table 2. Before data collection, the research team will be trained in standard operational procedures through online meetings with the assistance of tutorials and videos.

**Table 2:** Schedule of study activities

Measure	Screening	Baseline	12 months	24 months
Informed consent form	x			
Eligibility criteria screen	x^(a)^			
Sociodemographics	x^(a)^			
Medical history	x			
International Physical Activity Questionnaire		x^(b)^		
Mini-Mental State Exam		x^(b)^		
7-point subjective global assessment		x^(b)^	x^(b)^	
SARC-F and SARC-CalF		x^(b)^	x^(b)^	
***Primary outcomes***				
Handgrip strength		x^(c)^	x^(c)^	
Five-time sit-to-stand test		x^(c)^	x^(c)^	
4-m gait speed		x^(c)^	x^(c)^	
Muscle mass		x^(d)^	x^(d)^	
***Secondary outcomes***				
Falls			x	x
Hospitalization			x	x
Mortality			x	x

^a^After signing the informed consent form

^b^The application of questionnaires will be conducted during hemodialysis session

^c^Assessment of physical function will be conducted before hemodialysis and the mid-week session

^d^Assessment of body composition will be conducted after hemodialysis and the mid-week session

As shown in Table 2, both physical function and body composition assessments will be performed in a mid-week dialysis session. However, in the impossibility of carrying out all assessments on the same day, we will recommend the assessments to be taken place in the same week to avoid missing data.

### Covariates

Sociodemographic characteristics, health habits, clinical, and laboratory parameters will be collected through electronic medical records (Table 3). If necessary, the patient will be consulted to provide additional information.

**Table 3:** Operational definition of sociodemographic and clinical-laboratory variables

	**Collect form** (options of response)
**Demographic**	
Gender	Medical records (male or female)
Age	Medical records (in year)
Ethnicity	Medical records or self-reported (white, black, Asian, brown, and indigenous)
Smoking habit	Self-reported (never smoked, former smoker, current smoker)
Drinking habit	Self-reported (never consumed, former consumer, current consumer)
**Social**	
School degree	Self-reported (middle or high school, graduated, or post-graduated)
Marital status	Self-reported (married, single, divorced, widowed)
Salary range	Self-reported (number of minimum wages)
**Vascular access**	Medical records (arteriovenous fistula, catheter, or prosthetic graft)
**Clinical characteristics**	
Height	Medical records (in meters)
Dry body weight	Medical records (in kilograms)
Hemodialysis vintage	Medical records (in months)
Comorbid conditions	Medical records or self-reported (yes or no)
Etiology of chronic kidney disease	Medical records (confirmed diagnosis)
Medications	Medical records (no. of medications)
Physical rehabilitation	Professional standard report (yes or no, modality, frequency)
**Dialysis regiment**	Medical records (conventional, short-daily, hemodialysis, or hemodiafiltration)
**Laboratory variables**	Medical records

### Questionnaires

#### Physical activity

The International Physical Activity Questionnaire (IPAQ) - short version - will be used to assess the physical activity levels [27]. The IPAQ is composed of questions about activities at work, locomotion, sports and leisure, physical exercise, and activities of daily living performed over the past seven days. The frequency and duration of the activities will be evaluated in days and minutes, generating a total value in metabolic equivalent of task per week (MET-min/week). Patients will be classified according to their final sum of MET-min/week score: (a) low (< 600); (b) moderate (600–2999), and (c) high (≥ 3000) [28].

### Nutritional status

Nutritional status will be evaluated by the 7p-SGA. The 7p-SGA is recommended by the National Kidney Foundation Dialysis Outcome Quality Initiative (KDOQI) as a valid and reliable tool for assessing nutritional status in CKD [29], and it has been translated and validated into the Brazilian-Portuguese language [30]. This questionnaire is comprised of six components: weight change, dietary intake, gastrointestinal symptoms, functional capacity, comorbidities, and physical exam. The patients will be classified as having severe malnutrition (1 – 2 score), mild-moderate nutritional status (3 – 5 score), and good nutritional status (6 – 7 score) by an experienced renal dietitian.

### Cognitive status

The Mini-Mental State Examination (MMSE) will be used to evaluate cognitive status [31]. Patients will be classified into “normal cognitive status” and “cognitive impairment” according to specific cut-off scores based on their educational level adjusted to the Brazilian population [32].

### Muscle strength assessment

#### Handgrip strength

Muscle strength will be assessed through handgrip strength (HGS) with a hydraulic hand-held dynamometer before the dialysis session. Patients will seat with the shoulder in a neutral position with elbows flexed at 90◦ to the body position. Both arms will be assessed alternately, and three measurements will be recorded with a 1-min rest period. We will discard the first attempt as a “warm-up/familiarization” session, and the highest isometric strength during five seconds over the last two attempts will be recorded and expressed in kilograms (kg) [33]. Patients will receive verbal encouragement during evaluation. According to the EWGSOP2, low HGS is defined as < 27 kg for men and < 16 kg for women [34].

### Five-time sit-to-stand test

To assess the muscle strength of the lower limbs, we will apply the five-time sit-to-stand test (STS-5) before the dialysis session. For this, a 45-cm high chair will be used. Patients should standget up and sit in the chair five times with their arms crossed over their shoulders quickly as possible [35]. A verbal command of “Go!” will be said to start the test and started time and finished until the final standing position at the end of the fifth repetition. Patients will perform two attempts and the shortest time in seconds will be considered for analysis. A 1-min will be given between attempts. Low muscle strength of lower limbs will be defined as > 15 s to perform the test for both genders [34].

### Physical performance assessment

#### Gait speed

Physical performance will be evaluated by 4-m usual gait speed (GS) test before the dialysis session. Patients will be asked to walk on a course of 4-m at their usual pace, without running. There will be three attempts with an interval of up to one minute between attempts. We will discard the first attempt as a “warm-up/familiarization” session, and the shortest time between the two attempts will be recorded. Low GS will be defined when the patient has a time ≤ 0.8 m/s to complete the entire distance for both genders [34].

### Body composition and anthropometric assessment

Dry body weight (kg) and height (meters) will be collected via medical records. Body mass index (BMI) will be calculated using the formula dry body weight/height^2^.

As markers of muscle mass, two measurements of the calf circumference (CC) on the right lower-limb will be performed using an inelastic and inextensible measuring tape with patients in a standing position [19]. The average of the two measurements will be considered for further analysis. Specific cut-off points according to gender will be used, and low CC will be ≤ 34 cm for men and ≤ 33 cm for women [19].

In addition, for exploratory analysis, the mid-arm muscle circumference (MAMC) will be used as a second marker of muscle mass. The arm circumference (in cm) will be measured with an inelastic and inextensible tape measure at a midpoint between the acromion and the olecranon. The triceps skinfold thickness will be measured with a skinfold caliper (Lange Skinfold Caliper^®^) with a precision scale of ± 1 mm, at the same point on the arm circumference using standard techniques. Arm circumference and triceps skinfold will be measured on the opposite side of the arm with the arteriovenous fistula or standardized on the right side for those with a catheter after the dialysis session. The measurements will be taken three times and the average will be considered for further analyses. MAMC will be calculated as proposed by Frisancho (1981) [36]:

$$\mathrm{MAMC}\;(\mathrm{cm})=\mathrm{arm}\;\mathrm{circumference}\;(\mathrm{cm})-\left(0.314\;\mathrm X\;\mathrm{triceps}\;\mathrm{skinfold}\;(\mathrm{mm})\right)$$

Standard percentages of MAMC are obtained using reference values from National Health and Nutrition Examination Survey percentile distribution tables by Frisancho [36].

Finally, if the dialysis center has a Body Composition Monitor (Fresenius Medical Care), body composition can be evaluated, and sub-analyses will be further explored. Patients will be asked to remove all metallic objects. The procedure will be performed a minimum of 30 min or more after the end of the hemodialysis session to allow for redistribution of body fluids as recommended by the KDOQI by a dietitian or experienced researcher team staff [29]. Patients will remain in the supine position, with arms slightly abducted in relation to the trunk and legs slightly apart. Surface electrodes will be placed on the right side of the body on the dorsal surface of the hands and feet. However, those patients who have a pacemaker, metallic prostheses, or any amputation will not be evaluated. Appendicular muscle mass will be estimated using the equation by Sergi et al. [37]. This equation has demonstrated high accuracy with DEXA as a reference method [38]. According to the EWGSOP2, low muscle quantity is defined as < 7.0 kg/m^2^ for men and < 5.5 kg/m^2^ for women [34]. Each coordinated researcher will be advised that the same experienced evaluator conduct all muscle mass assessments after the dialysis session.

### Laboratories parameters

Data of albumin, calcium, C-reactive protein, creatinine, ferritin, glucose, glycated hemoglobin (HbA1c), Kt/V, parathyroid hormone, phosphorus, potassium, sodium, vitamin B12 and vitamin D (25-hydroxy) from blood samples will be collected via electronic medical records at baseline.

### Risk of sarcopenia assessment

#### SARC-F

To assess sarcopenia risk, we will use the SARC-F questionnaire and SARC-CalF. The SARC-F was previously translated into the Brazilian-Portuguese language [14]. The SARC-F questionnaire is comprised of five components: strength, walking ability, rising from a chair, stair climbing, and previous falls. Each component adds 0 – 2 points, and the total score ranges from 0 − 10; a score of 0 represents the better condition and a score of 10 represents the worst [13].

### SARC-CalF

SARC-CalF uses an additional measurement of muscle mass through the calf circumference (CC) to improve SARC-F prognostic capacity. To maintain concordance with the cut-off of low muscle mass, we will use  the same cut-off of ≤ 34 cm for males and ≤ 33 cm for females [19]. In presence of low CC, additional 10 points in the total score of SARC-F are inserted. Therefore, the total SARC-F + CC (SARC-CalF) scores range from 0 – 20 points. The scores between 0 − 10 are considered “no suggestive signs of sarcopenia at the time,” whereas scoring 11 − 20 points is considered “suggestive of sarcopenia”. Patients with a total score in SARC-F ≥ 4 points and SARC-CalF ≥ 11 points will be considered at sarcopenia risk [14].

### Operational diagnoses of sarcopenia, obesity, and sarcopenic obesity

**Fig. 4:**
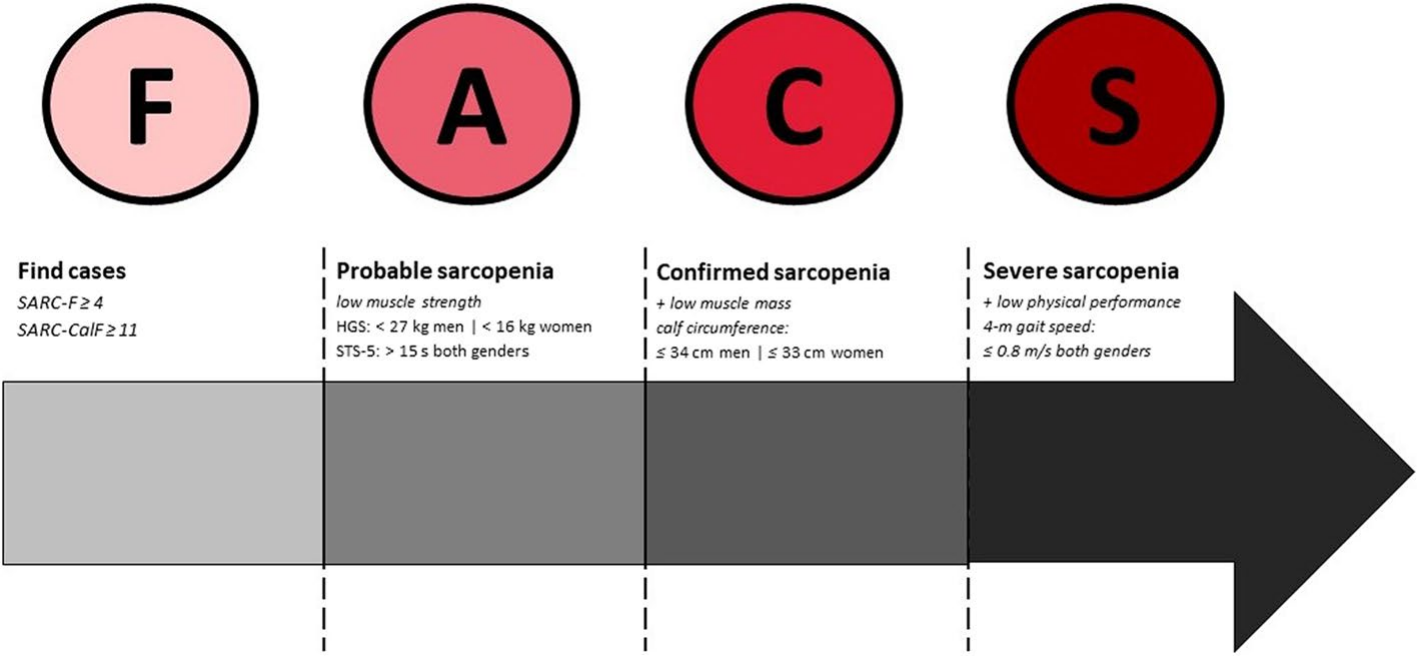
Algorithm for the diagnoses and severity of sarcopenia according to the EWGSOP2 (Adapted from Cruz-Jentoff et al. 2019)

In the SARC-HD study, we will follow the steps pathway proposed by EWGSOP2 as F-A-C-S (Find-Assess-Confirm-Severity; Fig. 4). Obesity will be defined as BMI ≥ 30, and fat mass index (kg/m^2^) > 9 and > 13 for men and women, respectively [39, 40]. Sarcopenic obesity will be defined as the coexistence of sarcopenia and obesity.

### SARC-HD study committees

Committees will be established to support principal investigators and coordinators. The steering committee will be responsible for the general supervision of the study, consensus meetings, staff training and standardization of methods, recruitment of additional centers, development of standard operational procedures, grant proposal, and analysis of scientific productions. The data management committee will be responsible for data analysis and periodic supervision of data accuracy and quality that will be reviewed regularly. Moreover, frequent training of study personnel will be conducted to improve data collection, such as frequent standardization of techniques to improve the quality, precision, and reliability of assessments.

### Data entry and monitoring data quality of dialysis center

All data will be collected by the local research teams and entered into an online spreadsheet (Google^®^) with individual passwords, hosted at the University Center ICESP. Periodically, the data management committee will independently verify and audit data, to reduce possible missing data, errors, and other cases, improving the quality, accuracy and assurance of collected data. Additionally, we will conduct online meetings via Google^®^ Meet with all study sites to provide feedback and assist in data and management collection.

### Ethical considerations

The study was approved by the Institutional Ethics Committe of the University Center ICESP on May 19, 2022 (no. 5.418.365) and complies with the Declaration of Helsinki. Of note, with the addition of new dialysis centers, addendums to the original protocol will occur. However, if necessary, the study protocol will be reviewed by other committees.

### Protocol amendments

Substantive modifications to the study protocol that impact the original proposal, which include changes in study design, eligibility criteria, procedures, planned analysis and/or additions will be first discussed among the steering committee, and the Institutional Ethics Committee may be notified. Minor administrative changes that did not impact the study and were previously agreed upon by the steering committee will be documented as a memorandum.

### Data dissemination, accessibility, authorship and sharing

Findings from the SARC-HD study will be reported in several ways. All manuscripts will be submitted to peer-reviewed scientific journals. Moreover, social media will be also used to disseminate findings to the public (@SARC_HD on Twitter^®^ and Instagram^®^). Guidelines and criteria of authorship were previously developed by the steering committee, which establishes all aspects of the study as evaluation and deliberation of requests for scientific productions and the role of principal investigators based on the International Committee of Medical Journal Editors. Requests for access to non-identified data should be made directly to the principal investigators only after four years of the end of the study.

### Statistical methods

#### Descriptive analysis

Sphericity, homoscedasticity, and normality of data will be tested before inferential analysis. For categorical variables, numbers and percentages within each category will be presented. Continuous variables will be presented as mean and standard deviation or median and interquartile ranges (25th and 75th percentiles). Patients will be stratified according to sarcopenia stages (e.g., non-sarcopenia, probable, confirmed, and severe) and descriptive statistics will be calculated separately. Lastly, no multiple imputations of outcome variables will be performed.

### Comparisons inter and intra-groups

Trajectories among the stages of sarcopenia will consider the exposures of interest in the present study. To compare the sarcopenia-related continuous variables the analysis of variance (ANOVA) of repeated measures and Tukey's Post hoc will be applied. If the assumptions are not met, the Kruskal–Wallis test will be used. Comparisons of proportions will be performed using the chi-square or Fisher's exact test. To evaluate the differences for the main dependent variables from baseline and 12 follow-up months among groups, the Two-way ANOVA will be applied to verify the time-group effect.

### Predictors of trajectories among sarcopenia stages

The transitional occurrence among sarcopenia stages, falls, hospitalization, mortality, and loss of follow-up will be first quantified and displayed using an alluvial plot. For the predictive analysis test, the bivariate logistic regression will be used to identify the predictors (independent variables) of trajectories from no sarcopenia to probable sarcopenia, confirmed sarcopenia, and severe sarcopenia (dependent variables), respectively. Additionally, the same statistical test will be applied to identify predictors of transition from the “severe sarcopenia” stage to the earlier stages (*i.e.*, confirmed, probable, and no sarcopenia). Uni and multivariate logistic regressions will be conducted to explore predictors of trajectories. In multivariate analysis, variables statistically significant in the univariate model, as well as clinically relevant variables, will be included. Other sensitivity analyses with further covariates will be conducted. All covariates inserted in the model will be based on the baseline moment. Patients who have not completed reassessments for sarcopenia traits at end of follow-up will not be included in the longitudinal analyses, being considered loss of follow-up.

### Association between sarcopenia and adverse clinical outcomes

To estimate the survival curves for each event (falls, hospitalization, and death), according to each sarcopenia stage and its traits, the Kaplan–Meier method and log-rank test will be used. Cox proportional hazard regression models will be conducted separately to analyze the associations of the sarcopenia stages and its traits (such as dichotomous categorical variables) with the incidence of events. The variables significantly associated with each adverse event in univariate analysis and also those with biological plausibility will be included in the adjusted models. The proportional hazard assumptions will be checked by Schoenfeld residuals. Moreover, the association of the SARC-F questionnaire, SARC-CalF and 7p-SGA with the adverse clinical outcomes will be explored. Results will be reported as hazard ratios (HR) with 95% confidence intervals (CI).

### Association and agreement of SARC-F, SARC-CalF and 7p-SGA with sarcopenia

To evaluate the association of SARC-F, SARC-CalF and 7p-SGA scores (continuous data) with the physical function tests (continuous data), the Pearson’s or Spearman’s rank correlation coefficient, according to the normality of variables, will be used. Binary logistic regression will be used to verify whether SARC-F, SARC-CalF and 7p-SGA as continuous and categorical variables (using previous cutoff points proposed) can be risk factors for different sarcopenia stages and their respective traits (dichotomous). The level of agreement of SARC-F, SARC-CalF and 7p-SGA with sarcopenia stages and their traits will be verified using the Kappa agreement coefficient [41]. Finally, receiver operating characteristic (ROC) curves will be generated to verify the diagnostic performance of screening tools, with the area under the curve (AUC) indicating the sensitivity, specificity, and positive and negative predictive values of the cuto-ffs proposed for SARC-CalF (≥ 11), SARC-F (≥ 4) and 7p-SGA to identify sarcopenia stages and its traits. The comparison of the difference between the ROC curves will be performed by the DeLong method [42].

### Sensitivity analysis

The associations between the delta (%, Δ) of baseline and 12-month values for the main dependent variables with adverse clinical outcomes will be verified using Cox models. Comparisons between survivors and non-survivors along the court will be made. Finally, to compare survival curves according to each adverse event among sarcopenia stages, dialysis modality (HD vs. hemodiafiltration), gender (male vs. female), and sarcopenic obesity vs. non-obesity and sarcopenic among other comparisons will be performed using the Log-rank test.

All analysis will be performed using the RStudio program (version 4.2.2, R Foundation for Statistical Computing, Vienna, Austria), Statistical Package for the Social Sciences (version 28.0, IBM Corp., Armonk, NY, USA), and STATA program (version 15, StataCorp LP, College Station, TX). The significance level will be pre-established at a *P-value* < 0.05 through bilateral analyses.

## Discussion

The observed deleterious impact of sarcopenia and its related traits in patients on HD underscores the need for a better comprehension of the determinants of sarcopenia in CKD, especially in those undergoing HD. Herein, we presented the design and detailed procedures that will be implemented in the SARC-HD study. In this sense, the SARC-HD study will provide novel findings. First, from a clinical setting perspective, we will use simple, fast, and low-cost tests that can be easily implemented as a routine in nephrology healthcare systems worldwide. Second, the screening and operational diagnoses of sarcopenia and monitoring of relevant clinical outcomes may serve as information for routine adjustments in dialysis centers, which commonly do not present this careful systematization. Furthermore, the development of novel and more effective strategies can be initialized from our findings. Therefore, we expect high-impact translational relevance to promote a better management of these patients.

## Conclusion

In summary, our ambition is that the SARC-HD study will clarify important knowledge gaps and identify potential determinants associated with sarcopenia and its progression, mainly contributing to advance knowledge translation to improve patients’ outcomes and guidance to support the implementation of sarcopenia screening in kidney care.

### Supplementary Information

**Additional file 1: Supplementary Material 1.** Full list of the SARC-HD Study center Investigators and Coordinators.

## Data Availability

The datasets generated during and/or analyzed during the current study are available from the corresponding author upon reasonable request.

## Abbreviations

7p-SGA: 7-Point subjective global assessment
BIA: Bioimpedance analysis
BMI: Body mass Index
CC: Calf circumference
EWGSOP2: Revised European Working Group on Sarcopenia in Older People
GS: Gait speed
HD: Hemodialysis
HGS: Handgrip strength
IPAQ: International physical activity questionnaire
KDOQI: National Kidney Foundation Dialysis Outcome Quality Initiative
MAMC: Mid-arm muscle circumference
MMSE: Mini-mental state exam
SARC-HD: SARCopenia trajectories and their association with clinical outcomes in patients on HemoDialysis
STS-5: Five-time sit-to-stand test
