# Supplementary material for: Design and methodology of the SARCopenia trajectories and associations with adverse clinical outcomes in patients on HemoDialysis: the SARC-HD study
Source: BMC Nephrol. 2023 Aug 15;24:239. doi: 10.1186/s12882-023-03168-4 (PMC10428584; doi:10.1186/s12882-023-03168-4)
Supplement: Supplementary file 1 — Additional file 1: Supplementary Material 1. Full list of the SARC-HD Study center Investigators and Coordinators. [file 12882_2023_3168_MOESM1_ESM.pdf]

Supplementary Material 1. Full list of the SARC-HD Study center Investigators and Coordinators

Centers (Federal District): 1) DaVita Advance, Brasília, Federal District
2) DaVita Águas Claras, Águas Claras, Federal District
3) DaVita Alvorada, Brasília, Federal District
4) DaVita Ceilândia, Ceilândia, Federal District
5) DaVita Gama, Gama, Federal District
6) DaVita JK, Taguatinga, Federal District
7) DaVita Asa Sul, Brasília, Federal District
8) DaVita Pacini, Brasília, Federal District
Principal Investigator: Heitor Ribeiro, PhD – heitor.ribeiro@icesp.edu.br
Coordinator: Marvery Duarte, MSc – marveryp@gmail.com
Collaborating researchers: Fábio Vieira – fabio.vieira.fisio@gmail.com; Jacqueline Monteiro, MD – Jacqueline.Monteiro-ext@davita.com; Priscila Varela – icr.priscila.fisio@gmail.com; Victor Baião, BSc – victor.baiao@aluno.unb.br; Ricardo Lima, PhD – professorricardomoreno@gmail.com; Antônio Inda-Filho, PhD – indafilho@gmail.com; Aparecido Ferreira, PhD – aparecido.ferreira@icesp.edu.br; Otávio Nóbrega, PhD – otavionobrega@unb.br

Center: NefroClass, Paulínia, São Paulo
Principal Investigator: Marco Uchida, PhD – uchida@unicamp.br
Coordinator: Dário Mondini – d233396@dac.unicamp.br
Collaborating researchers: Luiz Medina, MSc – luizmedina@hotmail.com; Luiza Sad, PT – luhsad@hotmail.com; Maria Gabriela Rosa, MD – mgabirosa@yahoo.com.br

Center: Renal Quality, Jundiaí, São Paulo
Principal Investigator: Marco Uchida, PhD – uchida@unicamp.br
Coordinator: Dário Mondini – d233396@dac.unicamp.br
Collaborating researchers: Luiz Medina, MSc – luizmedina@hotmail.com; Luiza Sad, PT – luhsad@hotmail.com; Flávio Nishimaru, MD – flavionishi@yahoo.com.br.

Center: Unidade de Diálise do Hospital das Clínicas da Faculdade de Medicina de Botucatu (HCFMB), Botucatu, São Paulo
Principal Investigator: Maryanne Zilli, PhD – maryanne.zilli@unesp.br
Coordinator: Maryanne Zilli, PhD – maryanne.zilli@unesp.br
Collaborating researchers: Fabiana Lourenço Costa - fl.costa@unesp.br; Isabele Carolina Rodrigues - isabele.rodrigues@unesp.br; Paula Torres Presti p.presti@unesp.br; Tabata Marinda Silva tabata.silva@unesp.br

Center: DaVita Bauru, Bauru, São Paulo
Principal Investigator: Clara Rosa, PhD – clarasuemi@hotmail.com
Coordinator: Henrique Disessa, BSc – henrique.disessa@unesp.br
Collaborating researchers: None.

Centers (Juiz de Fora): 1) DaVita Juiz de Fora, Juiz de Fora, Minas Gerais
2) DaVita Rio Branco, Juiz de Fora, Minas Gerais
Principal Investigator: Maycon Reboredo, PhD – maycon.reboredo@ufjf.br

Coordinator: Marina Silveira, RD – marinasp201821@gmail.com
Collaborating researchers:

Center: Clínica de Nefrologia de Araranguá, Araranguá, Santa Catarina
Principal Investigator: Daiana Bundchen, PhD - daiana.bundchen@ufsc.br
Coordinator: Daiana Bundchen, PhD - daiana.bundchen@ufsc.br
Collaborating researchers: Christine Zomer Dal Molin - christine.zdm@ufsc.br, Camila Rocha Vignali - camilavignali@gmail.com, Beatriz Rocha Viana - beatrizr.viana12@gmail.com, Karine Pires Costa - karinepires01@hotmail.com, Juliana dos Santos Raimundo - juliana.sr.torres@gmail.com, Laura Polo - laurapolo2001@gmail.com, Adriane Maria Horn - adrihorn@yahoo.com.br, Lucas Alves Pizzutti - lucas.pizzutti@icloud.com, Gabrielli Vieira Carrer - gabrielli.carrer@grad.ufsc.br, Laís Corrêa de Carvalho - correalais16@gmail.com, Josué dos Santos Barbosa Júnior - j.barbosa@grad.ufsc.br, Barbara Marjorie Schwabe - babi.schwabe@gmail.com, Rafaela Aguiar Rosa - rafaelaaguiarrosa14@gmail.com,

Center: Fundação Pró-Rim, Joinville, Santa Catarina
Principal Investigator: Bruna da R. Maggi Sant'Helena, PhD – Bruna.maggi@ielusc.br
Coordinator: Bruna da R. Maggi Sant'Helena, PhD – Bruna.maggi@ielusc.br
Collaborating researchers: Rodolfo Nunes Bittencourt - rodolfo.bittencourt@ielusc.br, Maria Cecília Kohler Panno maria.kohler@ielusc.br

Center: Unidade de Terapia Renal Jorge Bandarra Westphalen do Hospital São Vicente de Paulo, Cruz Alta, Rio Grande do Sul
Principal Investigator: Rodrigo Krug, PhD – rkrug@unicruz.edu.br
Coordinator: Rodrigo Krug, PhD – rkrug@unicruz.edu.br
Collaborating researchers: Paulo Ricardo Moreira, PhD - pmoreira@unicruz.edu.br, Thais Severo Dutra - thais.severo@hotmail.com, Jailton Possebom Marsola - jailtonpm17k@gmail.com , Anny Beatriz Somavilla- annysomavilla2002@gmail.com , Eduarda Martins Machado - eduarda1109@hotmail.com , Taiene Rodrigues - taienerodrigues1508@gmail.com , Amiria Teixeira Santana - lordamadeu00@gmail.com .

Center: Unidade de Hemodiálise do Hospital de Clínicas de Porto Alegre (HCPA), Porto Alegre, Rio Grande do Sul
Principal Investigator: Angélica Adamoli, PhD – aadamoli@hcpa.edu.br
Coordinator: Angélica Adamoli, PhD – aadamoli@hcpa.edu.br
Collaborating researchers: Catiussa Colling - ccolling@hcpa.edu.br, Rodrigo Jacobsen, Sabrina Rodrigues da Silva - srdsilva@hcpa.edu.br, Deise dos Santos Farias - dsfarias@hcpa.edu.br , Júlia Rodrigues - jborodrigues@hcpa.edu.br , Raíssa Teixeira - araissateixeira@gmail.com .

Center: Hospital Universitário São Francisco, Pelotas, Rio Grande do Sul
Principal Investigator: Maristela Bohlke, PhD – maristela.bohlke@ucpel.edu.br
Coordinator: Maristela Bohlke, PhD – maristela.bohlke@ucpel.edu.br
Collaborating researchers:
